# An investigation of the effects of infertility on Women’s quality of life: a case-control study

**DOI:** 10.1186/s12905-019-0805-3

**Published:** 2019-09-04

**Authors:** Katayoun Bakhtiyar, Ramin Beiranvand, Arash Ardalan, Farahnaz Changaee, Mohammad Almasian, Afsaneh Badrizadeh, Fatemeh Bastami, Farzad Ebrahimzadeh

**Affiliations:** 1 Department of Public Health, School of Health and Nutrition, Lorestan University of Medical Sciences, Khorramabad, Iran; 2 Public Health Center, 563 Hampshire Road, Apt 273, Westlake Village, CA 91361 USA; 3 Department of midwifery, School of Nursing and midwifery, Lorestan University of Medical Sciences, Khorramabad, Iran; 4 Department of the English Language, School of Medicine, Lorestan University of Medical Sciences, Khorramabad, Iran; 5 Department of Psychology, Lorestan University of Medical Sciences, Khorramabad, Iran; 6 Health Education and Promotion, Department of Public Health, School of Health and Nutrition, Lorestan University of Medical Sciences, Khorramabad, Iran; 7 Department of Biostatistics and Epidemiology, School of Health and Nutrition, Lorestan University of Medical Sciences, Khorramabad, Iran

**Keywords:** Infertility, Quality of life, WHOQOL-BREF questionnaire, Iran

## Abstract

**Background:**

Human instinctively desire to have offspring. Infertility can cause painful emotional experiences throughout the life mainly known as quality of life impairment. This study aimed to investigate the impact of infertility on a woman’s quality of life.

**Methods:**

A number of 180 infertile and 540 fertile women participated in this matched case-control study. The cases were selected through a combination of multistage stratified and cluster sampling methods. For each infertile woman three fertile women were randomly selected. The data gathering instrument consisted of demographic variables and the WHOQOL-BREF questionnaire. Data collection was conducted through interview with participants. The multivariate marginal model and SPSS software 21 were used for data analyses with a significance level of 0.05.

**Results:**

The results of the multivariate modeling show infertility can potentially affect various aspects of women’s quality of life such as physical health (*p* <  0.001), mental health (p <  0.001), social health (p <  0.001) and the total score of quality of life (p <  0.001) significantly.

**Conclusion:**

An infertile woman practice a relatively lower scores in QOL sub-scales of mental, physical and environmental health; while they experience a higher social health score than a fertile woman.

## Background

Reproduction is known as an essential human desire so that infertility may cause a great deal of psychosocial impairment [1]. According to WHO, infertility is defined as a disease of the reproductive system in which pregnancy does not occur after 1 year of continued intercourse [2]. Infertility is considered as a global concern which affects many aspects of life in both genders [3]. The rates even go up to 186 million people around the world [4]. About 10 percents of couples are currently suffering from infertility in Iran [5].

Infertility may work as a painful emotional experience [6, 7]. It can cause a lot of psychological issues including stress, anxiety, depression, diminished self-esteem, declined sexual satisfaction, and reduced quality of life [8–10]. The resulted psychosocial issues affect the female gender adversely more than her spouse [4], especially in societies where there are prejudices against women [9–11]. As such, an infertile woman may show a relatively high level of frustration and anger which affect her relationship with family, friends and even her spouse. Likewise, infertile women are more likely to develop mental illnesses, marital dissatisfaction, and impaired quality of life compared to the individuals of fertile group [9, 11, 12].

According to WHO, quality of life is a concept used to describe development, growth, and well-being which reflects individuals’ perceptions of their position in the community as well as their goals, expectations, standards, and priorities [13, 14]. Attitudes toward women’s infertility are often influenced by ethnic and cultural groups [15]. In the eastern societies, the community mainly expects women to play a role as a mother. This will cause many psychosocial concerns if pregnancy does not occur for any reason [16]. Therefore, more studies are required among eastern societies to reveal the impact of social, cultural and individual factors on an infertile woman’s quality of life [17].

Studying the quality of life among infertile women alarms the health authorities and subsequently let them spend a great deal of effort to help the infertile couples in one way or another [5]. There are already a few studies on the quality of life among infertile women in Iran; although those are largely descriptive and just follow a cross-sectional method which lacks a comparison group to analyze the impact of infertility on different aspects of life [5, 18, 19]. Most of these studies have been conducted using SF-36, a quality of life assessment questionnaire which evaluates the physical aspects of life quality [18]. There are multiple ethnical groups living in the country which requires researchers to run further studies in different regions as well. This study basically aimed to investigate the effect of infertility on a woman’s quality of life among population of Lorestan, Iran.

## Methods

### Study population and sampling methods

They were selected by means of a combination of multistage stratified and cluster sampling methods from population of Lorestan, Iran. We came up with a total of nine clusters. Each cluster contributes to a town in Lorestan, Iran. Five clusters (towns) were randomly selected out of them by sampling with varying probabilities; so that the more densely populated town, the higher chance of being selected.

There were two strata in each city for infertile women: The first stratum consisted of women who were being cared in a gynecology hospital or an infertility clinic, for which a non-probability consecutive sampling method was used. That means the information of an infertile woman was collected consecutively until the number of cases and their information were completed. The second stratum consisted of women who have been visited in a gynecology office. A total of 2--4 offices were selected in each geographical area using systematic random sampling method. We utilized a non-probability consecutive sampling method in each gynecologist office.

The control group consisted of fertile women who were matched for age, educational levels, and the duration of marriage with cases. For each infertile woman, three fertile women who met the matching criteria and lived in the same area were selected. In order to find the control subjects the investigators went to the same city block the infertile women were selected from. Then for each infertile woman they previously selected for the purpose of the study, they matched three fertile ones through a consecutive non-probability method. Data were gathered by trained interviewers.

The inclusion criteria for both groups comprised of giving consent for participating in the study, residing in the Lorestan province, as well as having monogamy with husband, lack of a psychological problems or an experience of stressful event related to the issue of infertility during the past 3 months, and no current use of alcohol or drugs.

*Infertility was defined as not being able to achieve pregnancy after 1 year of having regular, unprotected intercourse*. The inclusion criteria for the control group included no development of pregnancy during the course of study and a minimum gap of at least 4 months between the last given birth and the beginning of the study. A number of 120 fertile women were estimated to be suitable for the case group, however, considering the design effect; we had to select 180 individuals in the end. Since we matched three control subjects for each case, a number of 540 women were selected for the control group. The sample size eventually came up to 720 individuals.

The questionnaire consisted of two parts. First part of the questionnaire included demographic and background information of the participants such as age, occupational status, educational levels of the couple, duration of marriage, residential property ownership, address of residence, income, fertility and the status of spouse’s employment. The second part of the questionnaire consisted of the WHOQOL-BREF general measurement of life quality [20]. The internal consistency coefficient (Cronbach’s alpha) was evaluated and reported as satisfactory for all the sub-scales of the questionnaire, except for the social relation subscales (physical health dimension: α = 0.75, mental health dimension = 0.74, social health dimension = 0.70, and environmental health dimension = 0.75). We did not try to use SF-36 quality of life questionnaire for the purpose of our study because it only measures health-related quality of life but social and environmental health components of life quality [20].

### Statistical analysis

Frequency distribution tables, means, standard deviations and bar charts were used to describe the variables. Since individual-to-individual method of matching was used and the data was of a matched quadruplet type, the marginal model, and more specifically, the generalized estimating equations (GEE) method in parameter estimation was used for both univariate and multivariate data modeling. GEE is basically used to estimate the parameters of a generalized linear model with a possible unknown correlation between outcomes [21].

At first, the demographic and background variables between the fertile and infertile groups were compared through marginal model/GEE. In these GEE methods, a logit link function, along with exchangeable structure for covariance matrix was used. In each separate GEE, “Infertility status” was considered as dependent variable and a single demographic predictor was used as independent variable. We employed another marginal model/GEE to determine the relationship between quality of life scores and demographic variables. In these GEEs, an identity link function was deployed, and in each separate GEE, the quality of life score was considered as dependent variable while the only predictor was a single demographic variable.

The study was controlled for the effect of confounding factors. Since we aimed to investigate the impact of infertility on women quality of life, variables with a *P*-value of less than 0.25 in the aforementioned univariate approach were selected and entered into the multivariate model [22, 23]. Those demographic and background variables which were significantly associated with infertility and quality of life were considered as confounding variables.

For multivariate modeling, we utilized the identity link function along with an exchangeable structure for working correlation matrix in our GEE model. The quality of life scores and infertility status were considered as the dependent and independent variables, respectively. Confounding variables such as residential property ownership status, history of underlying diseases and consanguineous marriage were selected for the model. SPSS version 21 was used for data analyses with a significance level of 0.05.

## Results

We selected 180 infertile women and 540 fertile women from different cities of Lorestan, Iran for the purpose of our study. The mean age of cases and controls came up to 33.19 ± 5.9 and 33.11 ± 4.9, respectively (Table 1). Primary infertility was recognized as the most common reason for inability to reproduce (91.1%). The most frequent methods of treatment were IVF (45.6%) and medical therapy (43.8%). A proportion of 70.6% of cases and 69.4% of the controls were a housewife (Table 1). The prevalence of underlying diseases was higher among infertile women (20%) than the fertile ones (10.4%) (*P* = 0.023). Table 2 compares the demographic variables of cases to each dimension of women’s quality of life in Lorestan, Iran.

**Table 1:** Demographic and background variables among fertile and infertile women

Variable	Value	Fertile group	Infertile group	*P*-value ^b^
		Frequency (Percentage)	Frequency (Percentage	
Age range^a^	< 35	339 (62.8)	113 (62.8)	> 0.999
	≥35	201 (37.2)	61 (37.2)	
Duration of Marriage^a^	< 10	258 (47.8)	86 (47.8)	> 0.999
	≥ 10	282 (52.2)	94 (52.2)	
Educational Level^a^	Illiterate or primary school	156 (28.9)	52 (28.9)	> 0.999
	Junior high school to high school diploma	204 (37.8)	68 (37.8)	
	University	180 (33.3)	60 (33.3)	
Occupational Status	Housewife	375 (69.4)	127 (70.6)	0.794
	Employed	165 (30.6)	53 (29.4)	
Husband’s Educational Level	Illiterate or primary school	205 (38.0)	73 (40.6)	0.814
	Junior high school to high school diploma	152 (28.1)	49 (27.2)	
	University	183 (33.9)	58 (32.2)	
Husband’s Occupation	Unemployed	40 (7.4)	19 (10.6)	0.603
	White collar employee	126 (23.3)	38 (21.1)	
	Self-employed	296 (54.8)	99 (55.0)	
	Other	78 (14.4)	24 (13.3)	
Residential Property Ownership Status	Rented or living with parents	143 (26.5)	59 (32.8)	0.119
	Owned	397 (73.5)	121 (67.2)	
Having Underlying Diseases	No	484 (89.6)	144 (80.0)	0.023
	Yes	56 (10.4)	36 (20.0)	
Consanguineous Marriage	No	352 (65.2)	106 (58.9)	0.208
	Yes	188 (34.8)	74 (41.1)	
Type of Infertility	Primary	–	164 (91.1)	–
	Secondary	–	16 (8.9)	
Cost of Treating Infertility	< US$ 1500	–	35 (19.4)	–
	≥ US$ 1500	–	145 (80.6)	
Type of Infertility Treatment	Drug therapy	–	79 (43.8)	–
	Surgery	–	9 (5.0)	
	IVF	–	82 (45.6)	
	ICSI / IUI	–	10 (5.6)	

^a^ These variables were taken into consideration in matching the two groups

^b^ In these variables, only the data from the infertile group were used to assess relationships

The GEE method with a logit link fuction was used. In each separate GEE, “Infertility status” was considered as the dependent variable and each single demographic predictor was used as an independent variable

**Table 2:** Background variables by different dimensions of life quality

Variable	Range	Physical Health Dimension		Mental Health Dimension		Environmental Health Dimension		Social Health Dimension		Total score of quality of life	
		± ӿ s.d	*P*-value^a^	± ӿ s.d	*P*-value^a^	± ӿ s.d	*P*-value^a^	± ӿ s.d	*P*-value^a^	± ӿ s.d	*P*-value^a^
Age Range	< 35	50.0 ± 8.3	< 0.001	56. 2 ± 13.8	< 0.001	54.4 ± 9.9	0.315	33.8 ± 18.5	0.222	225.2 ± 27.9	0.509
	≤35	47.6 ± 8.0		51.9 ± 14.9		53.8 ± 9.4		36.2 ± 19.1		226.3 ± 27.4	
Duration of Marriage	> 10	50.8 ± 7.9	< 0.001	57.3 ± 13.3	< 0.001	54.6 ± 10.6	0.290	32.1 ± 18.4	< 0.001	223.6 ± 29.0	0.031
	≤10	47.6 ± 8.3		52.1 ± 14.8		53.8 ± 8.8		37.1 ± 18.8		227.5 ± 26.5	
Educational Level	Illiterate or primary school	46.8 ± 8.8	< 0.001	49.5 ± 14.3	< 0.001	52.3 ± 9.1	0.001	40.0 ± 18.8	< 0.001	228.5 ± 27.0	0.181
	Junior high school to high school diploid	48.8 ± 7.3		54.3 ± 13.5		53.5 ± 9.1		34.7 ± 18.9		225.3 ± 27.4	
	University	51.5 ± 8.3		59.3 ± 13.7		56.5 ± 10.4		30.2 ± 17.5		223.4 ± 28.6	
Occupational Status	Housewife	48.6 ± 8.4	0.014	52.9 ± 14.2	< 0.001	52.9 ± 9.4	< 0.001	36.4 ± 18.9	< 0.001	226.1 ± 28.1	0.334
	Employed	50.3 ± 7.8		58.6 ± 13.8		57.1 ± 9.8		30.7 ± 19.7		230.7 ± 28.4	
Husbands’ Educational Level	Illiterate or primary school	46.9 ± 8.4	0.001	50.0 ± 13.7	< 0.001	52.9 ± 9.1	< 0.001	40.6 ± 19.0	< 0.001	230.9 ± 28.8	< 0.001
	Junior high school to high school diploma	49.2 ± 7.7		54.8 ± 13.9		52.8 ± 9.0		32.3 ± 18.1		220.3 ± 24.7	
	University	51.6 ± 7.9		59.7 ± 13.6		56.8 ± 10.3		30.1 ± 17.4		223.8 ± 27.9	
Residential Property Ownership Status	Rented or Living with parents	48.1 ± 7.4	0.055	52.0 ± 13.1	0.002	51.8 ± 9.8	< 0.001	38.2 ± 18.3	0.024	225.9 ± 27.6	0.559
	Personally owned	49.7 ± 8.1		55.6 ± 14.7		55.1 ± 9.5		33.4 ± 18.8		226.5 ± 27.4	
Underlying Diseases	No	49.5 ± 6	< 0.001	55.9 ± 13.9	< 0.001	54.2 ± 9.7	0.850	33.3 ± 18.2	0.006	223.7 ± 27.5	< 0.001
	Yes	45.1 ± 8.4		45.8 ± 14.3		54.0 ± 9.3		44.7 ± 19.5		238.7 ± 25.6	
Consanguineous Marriage	No	49.4 ± 8.1	0.158	55.6 ± 13.9	0.035	54.2 ± 9.3	0.799	34.0 ± 17.5	0.089	225.3 ± 25.8	0.587
	Yes	48.6 ± 8.5		52.8 ± 14.9		54.2 ± 10.3		36.0 ± 20.9		226.2 ± 30.9	
Infertility Status	Fertile	50.2 ± 8.1	< 0.001	58.8 ± 12.0	< 0.001	54.3 ± 9.4	0.560	29.5 ± 15.7	< 0.001	219.9 ± 24.3	< 0.001
	Infertile	46.1 ± 8.1		41.9 ± 13.3		53.7 ± 10.4		50.6 ± 18.4		242.7 ± 30.4	
Type of Infertility ^b^	Primary	46.1 ± 8.1	0.771	42.0 ± 13.5	0.554	53.8 ± 10.4	0.723	50.7 ± 18.0	0.845	234.0 ± 30.4	0.601
	Secondary	45.3 ± 7.6		40.1 ± 11.1		53.5 ± 10.6		49.5 ± 22.5		239.2 ± 31.2	
Costs of infertility treatment ^b^	< US$ 1500	45.8 ± 6.0	0.820	47.0 ± 12.1	0.023	54.9 ± 11.3	0.401	44.3 ± 11.6	0.025	235.3 ± 24.4	0.128
	≥ US$ 1500	46.1 ± 8.4		40.6 ± 13.3		53.4 ± 10.2		52.1 ± 18.1		244.4 ± 31.5	
Type of Infertility Treatment ^b^	Medications only	46.8 ± 7.7	0.560	47.9 ± 12.2	< 0.001	55.5 ± 10.3	0.019	44.8 ± 18.4	0.005	238.9 ± 27.2	0.194
	Medications and surgery	47.2 ± 10.2		40.3 ± 14.3		45.5 ± 7.7		51.9 ± 13.0		233.4 ± 30.7	
	IVF	45.4 ± 7.8		37.7 ± 12.2		53.2 ± 9.8		55.7 ± 17.4		248.5 ± 30.9	
	IUI / ICSI	45.0 ± 10.4		29.6 ± 8.2		51.9 ± 13.8		52.5 ± 20.1		232.7 ± 43.1	

The GEE method with identity link fuction was deployed. In each separate GEE, quality of life scores was considered as dependent variable and each single demographic predictor was used as an independent variable

^a^ These variables were taken into consideration in matching the two groups

^b^ In these variables, only the data from the infertile group were used to assess relationships

Among infertile women, 52% of those who obtained a score of ≥70 on social dimension of quality of life were illiterate or had an educational level as of primary school. A proportion of 93% of infertile women were a housewife. Among infertile women, 67% of husbands were illiterate and 22% were unemployed. There was a significant difference between the mean scores of mental health in consanguineous and non-consanguineous married women (*P* = 0.01). The mean scores of both mental health and social health dimensions showed significant relationship with cost of treatment for infertility (*P* = 0.023) and (*P* = 0.025), respectively. There were also significant differences between the mean scores of mental, social, as well as environmental health dimensions and the method of treatment for infertility (P = < 0.001), (*P* = 0.005) and (*P* = 0.019), respectively (Table 2).

The results of the study showed that there is a significant statistical relationship between some of the independent variables and physical dimension of quality of life. For example; people aged 35 years or younger, those who had married for less than 10 years, women with an university educational level, individuals with no history of underlying diseases, as well as fertile and employed women had a higher score of physical dimension of life quality compared to the individuals of other categories (*p* <  0.05).

In addition, women younger than 35 years of age, those with an university educational level, individuals who were employed, people with no history of underlying diseases, women with an educated spouse, those with low costs of treatment for infertility, women who owned a house, as well as women with less than 10 years of marital life, those with no family marriage, fertile women, and infertile women under medical therapy only all had a higher average score of mental health dimension of quality of life compared to the individuals of the other subgroups (*p* < 0.05).

**Fig. 1:**
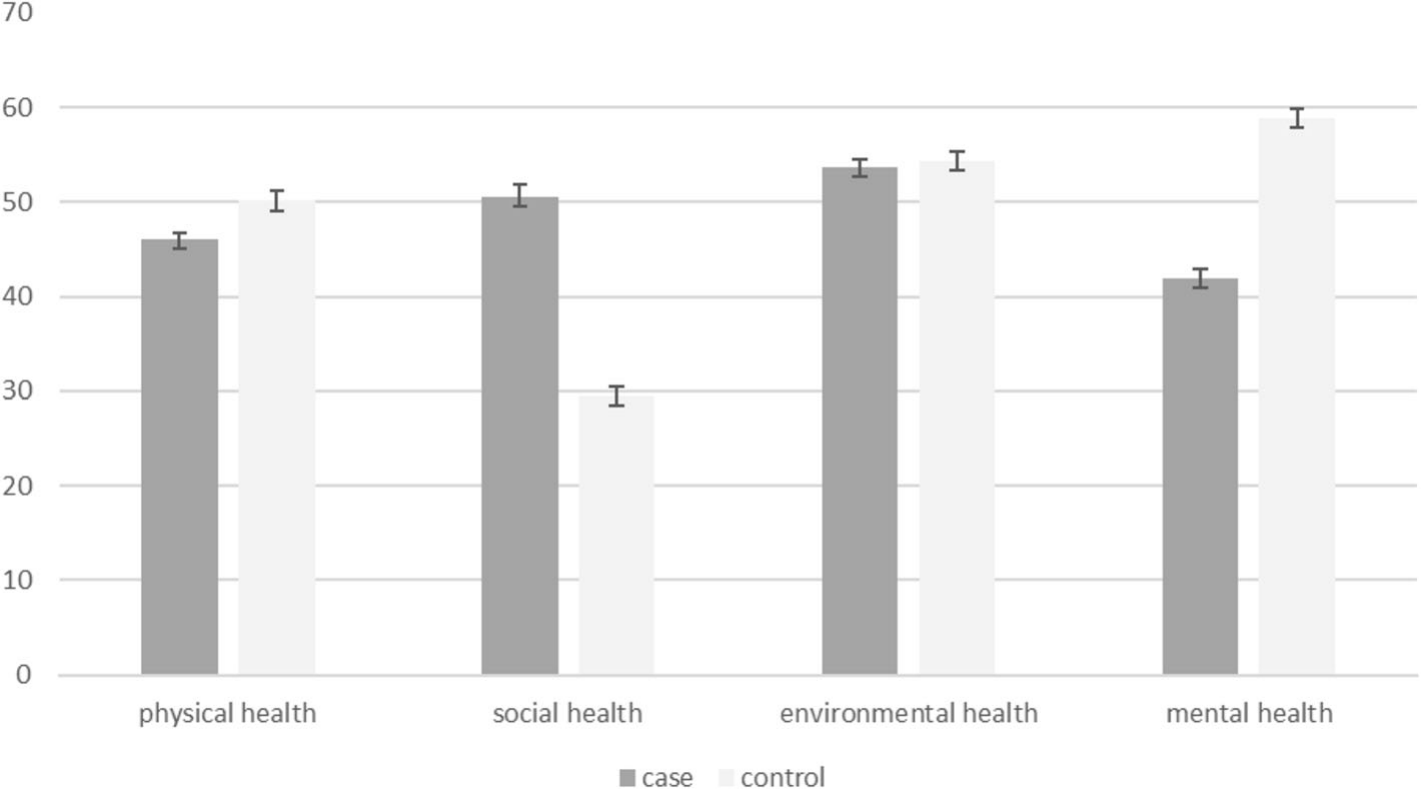
The mean scores of different dimensions of life quality among fertile and infertile women

Likewise, people who owned a house, those with an university educational level, women whose spouses had university education, employed women and infertile women under medical therapy only experienced a higher environmental health dimension of quality of life compared to the people of other categories (p < 0.05 for all). In addition, women with a marital relationship of over 10 years, undereducated or early school-age women, those whose spouses were not educated or just had elementary education, housewives, women living in permissive or paternal homes, infertile women, women with underlying illnesses, infertile women who suffered from a treatment cost of more than $ 1500 per month, and infertile women who received IVF treatment had a higher social dimension of quality of life score compared to the women of other categories (*P* < 0.05). Likewise, women with a marital relationship of more than 10 years, women whose spouses were undereducated or had elementary education, women with underlying diseases, and infertile women had a higher overall score of quality of life compared to the other categories (P < 0.05) (Table 2, Fig. 1).

Table 3 demonstrates the multivariate modeling for the impact of infertility on various aspects of women’s quality of life using the GEE method. Based on GEE 1 and 2 models which were analyzed both with and without adjustment for confounding variables, the effect of infertility on physical health dimension of life quality was significant (*P* < 0.001). After adjusting for confounding variables, the mean score of the physical dimension of quality of life among infertile women was 3.6 units lower than that of the fertile ones. Likewise, the effect of infertility on mental health dimension of quality of life was significant (*P* < 0.001). After controlling for confounding variables, the mean score of mental health dimension of life quality among infertile women was about 16.0 units less than that of the fertile ones.

**Table 3:** Multivariate modeling of the impact of infertility over different dimensions of life quality using GEE method

Variable	Model	Category	Estimated Regression Coefficient	Std. error	95% Confidence Interval	*P*-Value
Physical Health	Model 1^**a**^	Fertile	Referent			
		Infertile	−4.08	0.683	−5.418, −2.743	< 0.001
	Model 2^b^	Fertile	Referent			
		Infertile	−3.57	0.699	−4.941, −2.201	< 0.001
Mental Health	Model 1^**a**^	Fertile	Referent			
		Infertile	−16.96	1.037	−18.992, −14.928	< 0.001
	Model 2^b^	Fertile	Referent			
		Infertile	−15.95	1.043	−17.990, −13.903	< 0.001
Environmental Health	Model 1^**a**^	Fertile	Referent			
		Infertile	−0.60	0.838	−2.238, 1.046	0.477
	Model 2^b^	Fertile	Referent			
		Infertile	−0.39	0.837	−2.032, 1.249	0.460
Social Health	Model 1^**a**^	Fertile	Referent			
		Infertile	21.10	1.479	18.198, 23.994	< 0.001
	Model 2^b^	Fertile	Referent			
		Infertile	19.99	1.487	17.074, 22.903	< 0.001
The Total Score of Quality of Life	Model 1^**a**^	Fertile	Referent			
		Infertile	22.75	2.653	17.546, 27.946	< 0.001
	Model 2^b^	Fertile	Referent			
		Infertile	21.63	2.681	16.373, 26.881	< 0.001

^a^ Not adjusted for confounding variables

^b^ Adjusted for confounding variables

According to both GEE 1 and 2 models, the effects of infertility on the environmental dimension of life quality was insignificant (*P* = 0.477) and (*P* = 0.460), respectively. However, the impact of infertility on the social dimension of quality of life was found to be statistically significant (*P* < 0.001). After adjusting for confounding variables, the mean score of the social dimension of life quality among infertile women was 20.0 units more than that of the fertile ones. Finally, the effect of infertility on the total score of life quality was statistically significant (P < 0.001). After controlling for confounding variables, the mean score of life quality was 21.6 units more than that of fertile ones (Table 3).

## Discussion

The results of the current study showed an infertile woman experiences a relatively low quality of life by several dimensions in Iran. A few modalities of life quality such as physical, mental, and environmental health subscales scored lower among infertile Iranian woman than that of the fertile ones. Our research supports the findings of previous studies on this cause and effect relationship [24–26]. The social health dimension of life quality among infertile women however attained a higher score than that of the control group. This might have caused a large overall score of quality of life among infertile women.

According to the studies, Iranian women generally experience only an average overall health-related quality of life [27–29]. Nejat et al. was able to show that the mean score of Iranian women’s quality of life levels lower than that of other nation’s population of women in almost all sub-scales. The difference looked remarkable especially when physical and mental components of health came into the account [30]. Likewise, a study by Mirghafourvand showed a lower overall quality of life score among Iranian women than that of the Brazilian ones [27]. There are however studies that oppose the above findings which believe health-related quality of life among Iranian women scores higher than that of Turkish and Canadian ones [31, 32]. The difference in the results of the Iranian’s studies might be due to the diversity in socio-economic contexts, characteristics of the participants, sampling methods or a combination of all [27]. Likewise, use of different scales in these studies can cause difficulties comparing findings [33].

The findings of social health dimension in our study caused a significant difference in the overall score of the quality of life between groups. We found infertile women to have a higher social health score compared to the control group. This contradicts the results of previous studies [11, 19]; and might be due to the achieved score of ≥70 among 16% of infertile women. Likewise, the level of education and occupational status of women and their spouses did not match the distribution of education and occupation of the population. In this subgroup, a woman with a relatively low educational level played the role of a housewife, while she did not own a house; she enjoyed a greater social health. This might be due to the fact that an infertile woman receives more social support due to different reasons such as personal or familial relationships. In fact, an excellent social support can improve the physical and mental health; thus, it provides a relatively high social well-being and quality of life [34, 35].

The educational levels of couples and the occupational status of women predicted the quality of life in our study. According to the results, the educational status of a couple, women’s employment circumstances, and the status of ownership of a residential property affected a few dimensions of quality of life such as physical, mental, environmental and social health. As such, a couple with high educational level, an employed woman, and a homeowner enjoyed a better physical, mental, and environmental health. The results of few studies also indicate high educational level is associated with a high quality of life [36–39]. Therefore, low level of education can be linked to an increased probability of poverty, as well as a relatively low level of health, undesirable health behaviors, and an increased risk of mortality [27].

The results of the present study showed age range can affect the physical and mental health. Physical and mental health of the women younger than 35 was found to be significantly better than that of the women of older age groups. This is because a young woman has fewer physical and medical issues, more energy and ability to work, and higher self-esteem than an older one. A few studies have demonstrated a woman younger than 30 years of age experiences a better quality of life than an older woman [25, 33, 40]. A study of mental, environmental, and social health of women have brought up supportive results [41].

Duration of marriage can also affect the various dimensions of quality of life. Based on the findings, a woman experienced a relatively high physical, mental, and environmental quality of life within the first 10 years of marriage. Rostami et al. reported a woman in her first or second decade of marriage, while she is older; she is less satisfied with her marriage compared to a younger woman. This might be due to a negative assessment of physical appearance which adversely affects marital satisfaction. Therefore, it reduces a woman’s quality of life [34, 42, 43].

According to the present studies, having underlying illnesses can affect the various dimensions of life quality. As such, a woman with no underlying illnesses has a better physical, mental, and environmental health scores compared to an ill woman [24, 44, 45]. Proulex et al. was able to show that overall health had a significant relationship with almost all dimensions of quality of life [46]. Likewise, Maroufzadeh et al. showed infertile couples are more likely to have underlying illnesses such as anxiety (49.6%) and depression (33%) [12]. In fact, chronic diseases such as depression, diabetes, different types of cancer, etc., adversely affect those aspects of a woman’s quality of life which are related to overall health; thus, managing the above conditions may lead to a relatively better quality of life [27].

Our study has many strong points and we are perfectly confident in the validity of the results. The fact that it was a case–control study within cohort of Lorestan, Iran, enabled us to minimize the risk of selection bias. In addition, the design of the study allowed us to examine the link between infertility and quality of life from all socioeconomic classes. We were also able to examine a large number of variables as likely predictors of quality of life following failure to reproduction. Nonetheless, our study has a limitation as well. The fact that it is a non-longitudinal case-control study; we have had difficulty controlling it for some confounding variables. Therefore, prospective longitudinal studies are recommended for future studies on this link.

## Conclusion

Mental, physical, and environmental health components of quality of life may be adversely affected among infertile women, although the social health subscale may not. Other modalities such as educational attainment, employment, house ownership, and major illnesses also influence the quality of life. Given the fact that the quality of life among women of reproductive age affects the long-term health of each family member, health policy makers, family counselors, and psychologists are required to pay special attention to physical, mental, and environmental health dimensions of a woman’s life which adversely affects her quality of life.

## Data Availability

The datasets used and/or analyzed during the current study are provided by the corresponding author on a reasonable request.

## Abbreviations

GEE: Generalized Estimating Equations
IVF: In Vitro Fertilisation
SF-36: 36-Item Short Form Survey
WHO: World Health Organization
WHOQOL-BREF: WHO Quality of Life-BREF
